# PIPJ reconstruction after fracture including hemihamate transfer

**DOI:** 10.1186/1753-6561-9-S3-A45

**Published:** 2015-05-19

**Authors:** Amit Gupta

**Affiliations:** 1 Department of Orthopedic Surgery, University of Louisville, Kentucky, 40292, USA

## 

Proximal interphalangeal joint fracture dislocations are challenging injuries that can be managed in a wide variety of ways. The fracture dislocations of the PIP joints are classified by their mechanical stability and the percentage of joint involved in the fracture. Fracture dislocations that remain stable with less than 30 degree of flexion of the PIP Joint and those that have less than 20% of the articular surface involvement are generally considered stable and can be treated with extension block splinting.

The methods of treatment that will be discussed in this presentation include extension block splinting, traction of various kinds, pinning, and open reduction internal fixation.

When the PIP joint is very unstable and the fracture at the base of the middle phalanx so comminuted as to be unreconstructible, the state of the art management method is to use hemi hamate transfer that was described by Hill Hastings. This method of bringing stability to an unstable joint with the use of a unique osteochondral graft has revolutionized the management of PIP fracture dislocations. In this presentation, I will discuss the indications, detailed technique and outcomes of the hemi hamate transfer for fracture dislocations with appropriate case illustrations.

